# Pulp sensibility assessment following permanent tooth pulpotomy: a scoping review of clinical studies

**DOI:** 10.3389/fdmed.2026.1957091

**Published:** 2026-09-22

**Authors:** Cangül Keskin, Sıla Nur Usta, Özlem Sivas Yılmaz, Doğuş Bayraktar

**Affiliations:** 1 Department of Endodontics, Faculty of Dentistry, Ondokuz Mayis University, Samsun, Türkiye; 2 Department of Endodontics, Gülhane Faculty of Dentistry, University of Health Sciences, Ankara, Türkiye; 3 Department of Endodontics, Faculty of Dentistry, Bolu Abant İzzet Baysal University, Bolu, Türkiye

**Keywords:** cold test, electric pulp testing, heat test, vital pulp therapy (VPT), vitality

## Abstract

Vital pulp therapy (VPT), particularly pulpotomy, has emerged as a predictable treatment option for permanent teeth with deep caries and even in the presence of symptomatic irreversible pulpitis. Although numerous clinical studies have reported favorable outcomes, considerable variation exists in the methods used to assess postoperative pulp sensibility. This scoping review aimed to systematically map the pulp sensibility assessment methods, diagnostic methods, and follow-up schedules employed in clinical studies evaluating permanent tooth pulpotomy. This scoping review was conducted according to the PRISMA-ScR guidelines and prospectively registered in the Open Science Framework. Electronic searches were performed in PubMed (MEDLINE), Web of Science, and Scopus without date restrictions. Clinical studies involving partial or full pulpotomy in permanent teeth that reported postoperative pulp sensibility assessment were included. Data regarding study characteristics, pulpotomy procedures, sensibility assessment methods, follow-up intervals, and key findings were extracted and synthesized descriptively. The search identified 4,287 records, of which 35 studies fulfilled the eligibility criteria. The included studies comprised randomized controlled trials, prospective and retrospective clinical studies, and one quasi-experimental study. EPT combined with cold testing was the most frequently used assessment methods, followed by cold testing alone and EPT alone. Follow-up intervals varied substantially, ranging from 2 days to 8 years, although most studies evaluated sensibility at 6, 12, and 24 months. A consistent finding across studies was the markedly higher positive response rate obtained with EPT compared with cold testing after full pulpotomy. Teeth treated with partial pulpotomy generally maintained more favorable responses to both EPT and thermal tests. Several studies also reported transient postoperative thermal hypersensitivity during the early healing period, particularly following partial pulpotomy. Considerable heterogeneity exists in postoperative pulp sensibility assessment methods following permanent tooth pulpotomy. According to the included studies, EPT demonstrated higher response rates than cold testing after full pulpotomy. Standardized assessment methods and the incorporation of objective assessment methods are needed to improve the consistency of outcome reporting and facilitate comparisons among future VPT studies.

## Introduction

Dental caries remains a highly prevalent non-communicable disease globally, frequently driving patients to seek dental intervention due to pain and discomfort (1). As the carious lesion advances through the enamel and dentin, cariogenic bacteria and their by-products infiltrate the underlying dental pulp, eliciting an inflammatory response clinically diagnosed as pulpitis (2). Traditional clinical perspectives maintained that mature permanent teeth with deep caries and signs of symptomatic irreversible pulpitis (SIP) with normal apical tissues or symptomatic apical periodontitis, required full pulpectomy and root canal treatment (RCT), assuming that the unyielding dentinal walls and lack of collateral circulation rendered the inflamed pulp incapable of repair (3). However, this paradigm has been challenged by mounting clinical evidence. While histological findings indicate that severe inflammation and bacterial infiltration can be confined to the coronal pulp directly beneath the exposure (4), numerous clinical studies have demonstrated high success rates when treating SIP cases with pulpotomy (5–7). These outcomes confirm that the remaining pulp tissue often retains sufficient healing capacity to be successfully preserved.

This evolved understanding of pulpal pathobiology, coupled with significant advancements in bioactive restorative materials, has catalyzed a profound resurgence of interest in vital pulp treatments (VPT) (8). Far from being novel, these biologically based, minimally invasive strategies have experienced a modern renaissance, moving from historically unpredictable procedures to highly predictable alternatives to root canal therapy. VPT encompasses a spectrum of procedures aimed at preserving the health and immunological functions of the compromised dental pulp (9). Among these, partial and full pulpotomies have emerged as definitive treatments for permanent teeth. A partial pulpotomy involves the excision of 2–3 millimeters of the superficial inflamed pulp, whereas a full pulpotomy necessitates the complete amputation of the coronal pulp to the level of the root canal orifices (9). Following tissue removal and the achievement of hemostasis, a bioactive material, predominantly hydraulic calcium silicate-based cements (HCSCs) such as Mineral Trioxide Aggregate (MTA) or Biodentine is placed directly over the remaining pulp to seal the wound, induce hard tissue formation, and prevent microleakage (5). Driven by consistent reports of high clinical success, major dental organizations, including the European Society of Endodontology (ESE) and the American Association of Endodontists (AAE), have recently updated their clinical practice guidelines to endorse pulpotomy as a viable and highly successful alternative to RCT for mature permanent teeth with deep caries, even in the presence of SIP (9–11).

Despite the paradigm shift towards VPT and the favorable success rates reported in randomized controlled trials (RCTs), significant clinical and methodological challenges persist regarding the accurate assessment of post-operative pulpal status and the definition of clinical success (12, 13). Traditionally, endodontic diagnosis and post-operative monitoring have relied heavily on subjective neural sensibility tests, primarily thermal (cold/heat) tests and electric pulp testing (EPT) (Table 1). However, these modalities fundamentally measure the responsiveness of the A-delta and C nerve fibers, with the latter being a crucial indicator of severe pulpal inflammation and demise, without providing an objective assessment of the actual vascular blood supply, which is the true determinant of pulp vitality (14, 15). Furthermore, evaluating the outcome of a pulpotomy using sensibility tests presents a unique clinical dilemma. During a full pulpotomy, the entire coronal pulp is surgically excised. Consequently, post-operative sensibility testing must rely entirely on stimulating the remaining radicular innervation. However, this deeper radicular tissue may fail to respond to traditional thermal or electric stimuli due to the thick layer of insulating restorative material, the increased physical distance from the applied stimulus, and the loss of the superficially located coronal nerve plexus. This frequently renders these sensibility tests highly unpredictable as diagnostic tools for post-treatment monitoring (12, 16). Although objective vascular-based tools like Laser Doppler Flowmetry and pulse oximetry theoretically offer higher diagnostic accuracy for assessing true pulp vitality, their routine clinical application remains highly impractical due to significant technical limitations, high bias risks, and equipment accessibility constraints (17, 18).

**Table 1:** Characteristics of pulp sensibility and vitality assessment modalities.

Diagnostic method	Assessment type	Mechanism/Diagnostic target	Advantages	Limitations & Clinical applicability
Electric Pulp Testing (EPT)	Sensibility (Neural)	Delivers a direct electrical current to stimulate and depolarize A-delta nerve fibers within the pulp.	Demonstrates a higher proportion of positive responses post-pulpotomy. The electrical current appears to overcome the electrical resistance of insulating materials to stimulate radicular nerves (16).	Subjective. Cannot detect true vascular blood flow. Frequently yields false-negative results in immature teeth with incomplete root development (14, 21).
Cold Test (Thermal)	Sensibility (Neural)	Operates on the hydrodynamic theory. Thermal contraction causes dentinal fluid movement, which excites A-delta mechanoreceptors via intact odontoblasts (56).	The most universally accepted traditional method for detecting healthy or necrotic pulps in intact teeth. Elicits more consistent responses than EPT in immature teeth (14).	Prone to false negative responses due to the surgical amputation of the coronal odontoblast layer and the potent thermal insulation provided by thick layer of HCSCs.
Heat Test (Thermal)	Sensibility (Neural)	Stimulates slow-conducting, unmyelinated C-fibers via heat application, which are responsible for dull, poorly localized, and lingering pain.	Useful for replicating chief complaints in teeth with severe, lingering pain indicative of symptomatic irreversible pulpitis.	Highly prone to exaggerated false-positive responses during the transient acute inflammatory healing phase. Thermal insulation from thick biomaterials hinders stimulus transmission. Least utilized modality in clinical trials.
Laser Doppler Flowmetry (LDF)	True Vitality (Vascular)	Utilizes infrared light to directly measure dynamic pulpal blood flow and red blood cell velocity (57).	Diagnostic adjunct requiring further clinical validation. Completely objective and non-invasive. Does not rely on the patient's subjective pain response.	Clinically impractical. Highly sensitive to movement artifacts. Measurements are heavily restricted by enamel/dentin thickness and thick coronal restorations. High equipment cost.
Pulse Oximetry (PO)	True Vitality (Vascular)	Uses photometric methods to assess the oxygen saturation levels of the pulpal blood supply (57).	Diagnostic adjunct requiring further clinical validation. Objective and non-invasive indicator of actual tissue perfusion rather than nerve responsiveness.	Clinically impractical. Requires custom-made sensor holders to fit the tooth accurately. Readings are severely hindered by thick restorations and tooth discoloration.
Ultrasound Doppler Flowmetry (UDF)	True Vitality (Vascular)	Detects waveform pulsatile signals of pulpal blood flow utilizing ultrasound waves (58).	Experimental adjunct requiring further clinical validation. Non-invasive and radiation-free. Unlike LDF, it does not require calibration and can differentiate signals from pulp versus surrounding tissues.	Highly technique-sensitive. Readings are easily affected by probe angulation, poor adaptation, and the thickness of the overlying enamel/dentin.
Photoplethysmography (PPG)	True Vitality (Optical/Vascular)	A light source detects variations in blood volume within the microvascular bed synchronized with heart contractions (59).	Experimental adjunct requiring further clinical validation. Non-invasive and potentially low-cost (60). Shows low susceptibility to signal contamination from saliva or periodontal tissues compared to other optical methods.	Pulse signals can be obscured by movement or respiration artifacts. Highly calcified chambers or thick restorations limit light transmission.
Tooth Temperature (Thermometry)	Inflammatory Indicator	Measures tooth surface temperature (using laser-guided infrared thermometry) to evaluate pulpal tissue integrity.	Quick, non-invasive, and cost-effective (61). Vital teeth tend to demonstrate better surface rewarming capabilities.	Heavily influenced by periodontal tissue circulation, saliva flow, and ambient room temperature. Ineffective in teeth with extensive crowns or restorations.
Molecular Biomarkers	Inflammatory Level (Molecular)	Quantifies inflammatory mediators and cytokines from pulpal blood or dentinal fluid samples.	Diagnostic adjunct requiring further clinical validation. Offers the highest theoretical accuracy in staging pulpal inflammation (reversible vs. irreversible) at a cellular level and predicting treatment prognosis.	Promising but not yet routine. Currently requires laboratory analysis. Rapid chair-side test kits are still in development for routine dental practice.

While the literature contains an increasing number of clinical studies investigating various biomaterials and pulpotomy techniques, there remains an evident lack of methodological consistency and standardized reporting regarding how and when pulp sensibility is assessed post-operatively. Therefore, a comprehensive scoping review of the sensibility assessment methods employed in recent clinical studies is essential to highlight existing gaps in methodological reporting and to guide the standardization of future study designs. The primary aim of this scoping review is to systematically map, identify, and categorize the test modalities and follow-up timeframes used to assess pulp sensibility following partial and full pulpotomy procedures in permanent teeth.

## Materials and methods

### Protocol and registration

This scoping review was conducted following the Preferred Reporting Items for Systematic Reviews and Meta-Analyses Extension for Scoping Reviews (PRISMA-ScR) guidelines (19). The review protocol was prospectively registered on the Open Science Framework (doi: 10.17605/OSF.IO/Z2WN7).

### Eligibility criteria

The primary objective of this scoping review was to comprehensively map the existing evidence on pulp sensibility assessment following tooth pulpotomy. To achieve this, the Population, Concept, and Context (PCC) mnemonic, as recommended by the Joanna Briggs Institute, was used to establish the eligibility criteria and guide the study selection process (20). According to this framework, the population comprised permanent teeth treated with pulpotomy, while the concept focused on the methods used for pulp sensibility assessment (e.g., cold test, EPT). Finally, the context encompassed the follow-up evaluations performed after these pulpotomy procedures.

Studies including randomized clinical and controlled trials, retrospective and prospective clinical studies, quasi-experimental studies evaluating pulp sensibility following pulpotomy in permanent teeth (immature or mature) were considered eligible for inclusion. Studies had to involve either partial or full pulpotomy and report at least one postoperative follow-up assessment of pulp sensibility. Eligible studies were required to clearly describe the method(s) used for pulp sensibility assessment during follow-up and report the corresponding findings. No restrictions were placed on the pulpotomy material or the clinical indication for pulpotomy. Moreover, studies were excluded if they did not clearly report the pulp sensibility test(s) used during postoperative follow-up, involved pulpotomy procedures performed in primary teeth, or were histological or animal studies. Case reports and case series, review articles, editorials, conference abstracts, or other non–peer-reviewed publications were also excluded. Furthermore, studies with unavailable full texts were excluded if the articles could not be obtained after attempts to contact the corresponding author.

### Search strategy and information sources

A comprehensive search strategy was developed by two investigators (SNU and CK) experienced in endodontic research and evidence synthesis. Relevant keywords and Medical Subject Headings (MeSH) were combined using the Boolean operators “AND” and “OR” to optimize the retrieval of eligible studies. Electronic searches were conducted in PubMed (MEDLINE), Web of Science (all databases), and Scopus on June 18, 2026. Additionally, a manual search of the grey literature was undertaken to identify potentially eligible studies not retrieved through the electronic database searches. Only written in English language articles were searched without any date limit. The detailed search strategies are presented in Table 2, while the study selection process is summarized in Figure 1.

**Table 2:** Search strategy and obtained articles.

Search set	PubMed	Web of Science	Scopus
#1	(((((((pulpotomy[MeSH Terms]) OR (partial pulpotomy)) OR (total pulpotomy)) OR (full pulpotomy)) OR (complete pulpotomy)) OR (cvek)) OR (vital pulp treatment)) OR (vital pulp therapy) (No. of articles = 4,469)	(((((((ALL=(pulpotomy)) OR ALL=(partial pulpotomy)) OR ALL=(total pulpotomy)) OR ALL=(full pulpotomy)) OR ALL=(complete pulpotomy)) OR ALL=(cvek)) OR ALL=(vital pulp treatment)) OR ALL=(vital pulp therapy) (No. of articles = 4,579)	ALL (“pulpotomy”) OR ALL (“total pulpotomy”) OR ALL (“partial pulpotomy”) OR ALL (“Full pulpotomy”) OR ALL (“complete pulpotomy”) OR ALL (“cvek”) OR ALL (“vital pulp treatment”) OR ALL (“vital pulp therapy”) (No. of articles = 18,664)
#2	((((((((((((dental pulp vitality test[MeSH Terms]) OR (dental pulp sensibility test[MeSH Terms])) OR (electric pulp test[MeSH Terms])) OR (electric pulp tests[MeSH Terms])) OR (flowmetry, laser doppler[MeSH Terms])) OR (laser doppler flowmetry[MeSH Terms])) OR (cold test)) OR (heat test)) OR (thermal test)) OR (sensibility)) OR (vitality)) OR (EPT)) OR (pulp response) (No. of articles = 149,413)	((((((((((ALL=(dental pulp vitality test)) OR ALL=(dental pulp sensibility test)) OR ALL=(electric pulp test*)) OR ALL=(laser doppler flowmetry)) OR ALL=(cold test)) OR ALL=(heat test)) OR ALL=(thermal test)) OR ALL=(sensibility)) OR ALL=(vitality)) OR ALL=(EPT)) OR ALL=(pulp response) (No. of articles = 694,090)	ALL (“dental pulp vitality test”) OR ALL (“dental pulp sensibility test”) OR ALL (“electric pulp test”) OR ALL (“laser doppler flowmetry”) OR ALL (“cold test”) OR ALL (“heat test”) OR ALL (“thermal test”) OR ALL (“sensibility”) OR ALL (“vitality”) OR ALL (“EPT”) OR ALL (“pulp response”) (No. of articles = 302,356)
Summary	(#1 AND #2) = 923	(#1 AND #2) = 869	(#1 AND #2) = 2,495

**Figure 1:**
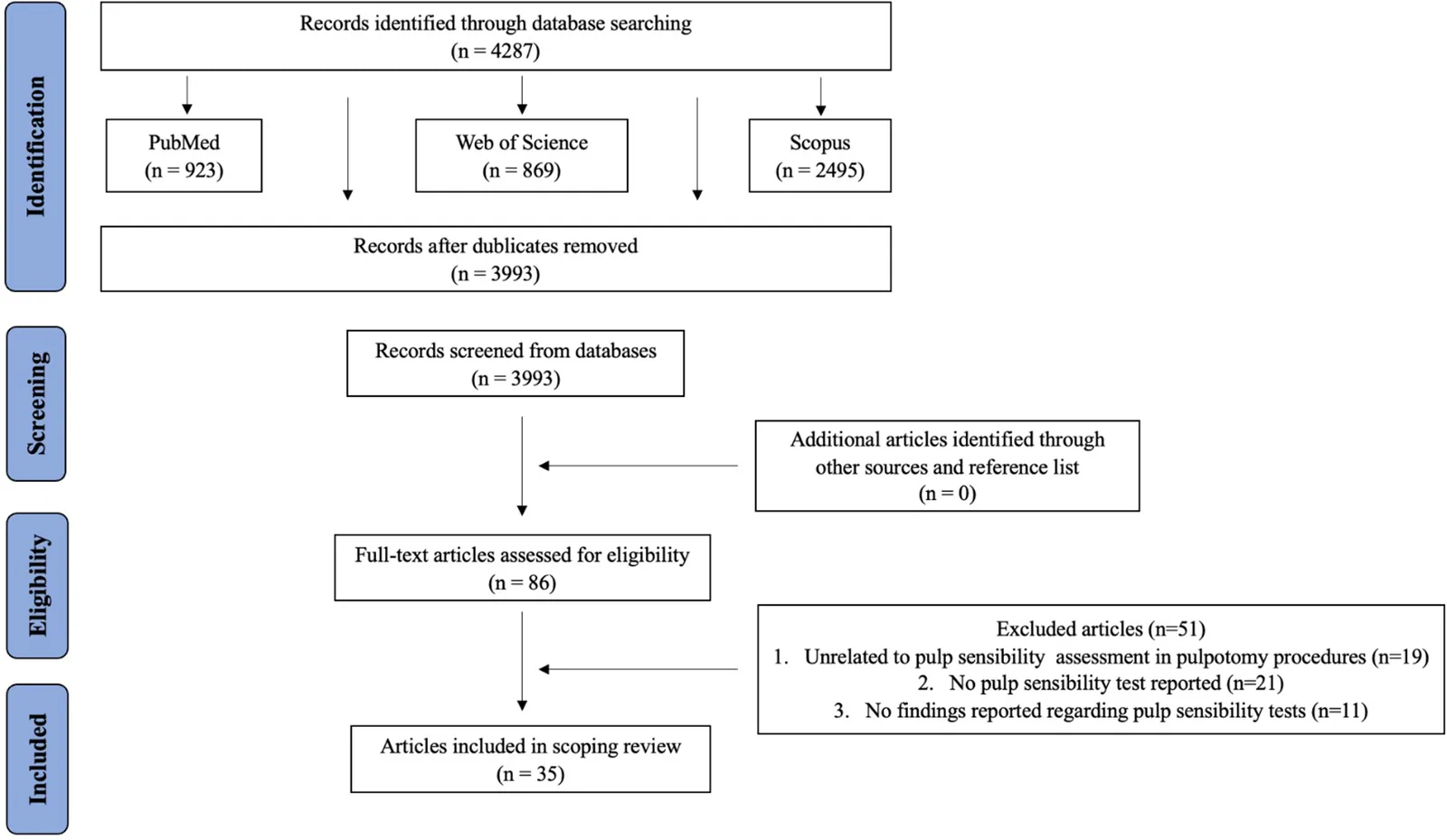
Flowchart of the searching process.

### Data charting process

All retrieved records were imported into Microsoft Excel (version 15.0; Microsoft, Redmond, WA, USA), where duplicates were identified and removed. Prior to the formal screening, the two investigators (SNU and CK) underwent a calibration exercise. They independently screened a random sample of 50 records to ensure a standardized interpretation and application of the predefined eligibility criteria. Any initial discrepancies were resolved through discussion to achieve a consensus. The main study selection process consisted of two stages: (1) title and abstract screening and (2) full-text eligibility assessment. Two investigators (SNU and CK) independently screened all records and subsequently assessed the full-text articles against the predefined eligibility criteria.

### Data items and synthesis of results

Relevant data were extracted from the included studies using a standardized data-charting form. The extracted information included bibliographic details (author, country, and year of publication), study design, characteristics of the study population, pulpotomy procedure, stage of root development, tooth type, pulp diagnosis, follow-up period, pulp sensibility test(s) used during follow-up, and the corresponding main findings.

The extracted data were synthesized descriptively to map the current evidence regarding the use of pulp sensibility tests following pulpotomy in permanent teeth. Owing to the heterogeneity of the included studies, a structured qualitative framework was adopted for data synthesis, in accordance with standard scoping review guidelines (e.g., JBI methodology). The studies were categorized according to: (1) pulpotomy procedure (partial or full pulpotomy), (2) tooth type (incisor, premolar, or molar), (3) preoperative pulp diagnosis (healthy pulp, reversible pulpitis, or irreversible pulpitis), and (4) sensibility test utilized (cold, heat, or EPT). These specific domains were established *a priori* based on existing endodontic literature, as they were considered clinically relevant sources of heterogeneity that could significantly influence the reported outcomes and diagnostic accuracy.

## Results

### Selection of sources of evidence

The literature search yielded 4,287 records across the electronic databases, comprising 923 from PubMed (MEDLINE), 869 from Web of Science, and 2,495 from Scopus. After removing duplicate records, 3,993 unique studies remained and were screened based on their titles and abstracts.

Following the initial screening, 86 articles were retrieved for full-text assessment. Eligibility was independently evaluated by the reviewers using the predefined inclusion and exclusion criteria. The inter-rater reliability for the study selection process was evaluated using Cohen's Kappa statistic, yielding a strong agreement score (κ = 0.85). Ultimately, 35 studies fulfilled the eligibility criteria and were included in the final synthesis of this scoping review (6, 13, 16, 21–52). Summary of the selected studies along with key findings is shown in Table 3.

**Table 3:** Summary of the selected studies, along with key findings.

Author, year, country	Title	Study design	Sample size	Age range	Tooth type	Pulp diagnosis	Type of pulpotomy	Sensibility assessment method	Time of assessment	Key findings
Wani et al., 2023 India (62)	Selective removal to soft dentine versus full pulpotomy for management of proximal deep carious lesions: A randomized controlled non-inferiority trial	A randomized controlled non-inferiority trial	*n* = 60	16–35	Molar	Reversible pulpitis	Full	Cold test and EPT	Single assessment: 6 months	Treatment outcome in 1 tooth from each of the two study groups was categorized as a failure (patients reported within 6 months with complaint of pain and teeth that did not respond to pulp sensibility tests)
Shallal-Ayzin et al. 2021 USA (22)	A prospective analysis of the correlation between postoperative pain and vital pulp therapy	Retrospective clinical study	*n* = 73	15–78	Anterior Premolar Molar	Healthy pulp	Partial	Cold test and EPT	Single assessment: 6 months Loss to follow-up = 22	The vital pulp treatments had a high success rate in maintaining pulp vitality.
Petrovic et al. 2023 Germany (23)	Outcome after partial pulpotomy: Long-term results of the prospective clinical MMP-9 study	Prospective clinical study	*n* = 18	19–74	Anterior Molar	Normal pulp and Reversible pulpitis	Partial	Cold test	Serial assessments: Annually (1 to 8 years). (Overall range: 2–8 years, mean: 4.4) Loss to follow-up = 1	All teeth responded the vitality test as positive.
Chua et al. 2023 Singapore (24)	One-year outcome of selective caries removal versus pulpotomy treatment of deep caries: A pilot randomized controlled trial	A pilot randomized controlled trial	*n* = 120	21–65	Incisor Canine Premolar Molar	Reversible pulpitis	Full	Cold test and EPT	Serial assessments: 6 and 12 months Loss to follow up = 7	4 teeth in the SCR group were being nonresponsive to sensibility tests at 6 months.
Careddu et al. 2021 Ireland (13)	A prospective clinical study investigating the effectiveness of partial pulpotomy after relating preoperative symptoms to a new and established classification of pulpitis	Prospective clinical study	*n* = 51	14–60	Premolar Molar	Irreversible pulpitis	Partial	Cold test	Serial assessments: 1 week, 3, 6, and 12 months Loss to follow-up = 10	1-year follow-up (Cold test): Reversible pulpitis: 13 positive, 5 unresponsive; Irreversible pulpitis: 14 positive, 5 unresponsive (Total: 10/41 unresponsive but clinically successful)
Rechitra et al., 2021 India (25)	Influence of occlusal and proximal caries on the outcome of full pulpotomy in permanent mandibular molar teeth with partial irreversible pulpitis: A prospective study	Prospective clinical study	*n* = 80	16–35	Molar	Irreversible pulpitis	Full	Cold test and EPT	Serial assessments: 6 and 12 months Loss to follow-up = 0	In both groups, greater numbers of teeth responded to EPT than the cold test. However, on comparing the response of EPT between occlusal and proximal caries group, no significant difference was observed.
Al-Batayneh & Abdelghani 2022 Jordan (26)	Outcome of vital pulp therapy in deeply carious molars affected with molar incisor hypomineralisation (MIH) defects: a randomized clinical trial	Randomized clinical trial	*n* = 50	6–16	Molar	Reversible pulpitis Irreversible pulpitis	Partial	Cold test	Serial assessments: 3, 6, 12, and 24 months Loss to follow up = 3	Upon presentation for treatment, 76% of the patients reported sensitivity which was reproduced by cold testing.
Mishra et al. 2024 India (27)	Outcome of novel pulp capping modalities after full pulpotomy in teeth diagnosed with irreversible pulpitis: A prospective randomized clinical trial	Randomized clinical trial	*n* = 174	15–45	Molar	Irreversible pulpitis	Full	Cold test and EPT	Serial assessments: 1 and 3 weeks, and 1, 3, 6, and 12 months Loss to follow up = 34	In case of pulp sensibility testing using EPT and cold spray, no response was recorded at 1 week, 3 week, 2 months, 3 months, 6 months, and 1 year.
Aravind et al. 2022 India (16)	Response to pulp sensibility tests after full pulpotomy in permanent mandibular teeth with symptomatic irreversible pulpitis: A retrospective data analysis	Retrospective clinical study	*n* = 120	16–35	Molar	Irreversible pulpitis	Full	Cold test and EPT	Retrospective single assessment: 12 months	A higher percentage of teeth (94.7%) responded to the EPT in comparison with the cold test (13.5%). Sex-based responses to the EPT and the cold test were similar. The age-based response was similar for the EPT but was significant for the cold test. The tooth quadrant (left and right), the tooth (mandibular first and second molar), and the location of restoration (proximal or occlusal) did not influence the response of either the EPT or the cold test. At the 1-year follow-up, all the teeth that were classified as clinically and radiographically successful responded to the EPT; however, only 13 teeth responded to the cold test.
Jassal et al. 2023 India (28)	Outcome of partial and full pulpotomy in cariously exposed mature molars with symptoms indicative of irreversible pulpitis: A randomized controlled trial	Randomized controlled trial	*n* = 50	>18	Molar	Irreversible pulpitis	Partial Full	Cold test and EPT	Single assessment: 12 months Loss to follow-up = 1	Pulp sensibility at 1-year follow up showed a positive response in 95.45% (21/22) cases in partial pulpotomy group and 63.6% (14/22) cases in full pulpotomy group when tested with EPT. Additional cold testing, 45.45% (10/22) in partial pulpotomy and 13.63% (3/22) in the full pulpotomy group showed a positive response.
Asgary et al. 2022 Iran (6)	Outcomes of root canal therapy or full pulpotomy using two endodontic biomaterials in mature permanent teeth: a randomized controlled trial	Randomized controlled trial	*n* = 157	14–60	Molar	Irreversible pulpitis	Full	Cold test and EPT	Single assessment: 24 months Loss to follow up = 10	The results of sensibility pulp tests at 2-year recall revealed that although only 2 cases in each pulpotomy arms had responded to the cold test, the number of cases responding to electric pulp test in the pulpotomy arms was statistically different.
Kazemipoor et al. 2018 Iran (29)	Evaluation of the response to electric pulp testing before and after pulp chamber pulpotomy	Prospective clinical study	*n* = 60	20–60	Premolar Molar	Irreversible pulpitis	Full	EPT	Single assessment: 2-to 5 days	The mean value of response to EPTwas 3.08 ± 0.8 before and 4.5 ± 1.6 after pulpotomy. The pulpal response to EPT before and after pulpotomy had significant difference
Rao et al. 2020 China (30)	Comparison of iRoot BP Plus and Calcium Hydroxide as pulpotomy materials in permanent incisors with complicated crown fractures: A retrospective study	Retrospective clinical study	*n* = 205	5–13	Incisors	Healthy pulp	Partial	EPT	Serial assessments: 6–8 weeks, 1 year, and yearly up to 12 to 24 months (Mean follow-up:17.5 ± 4.4 months) Loss to follow up = 50	In the iRoot BP Plus group only 1 immature tooth failed because of the presence of apical periodontitis supported by radiographic radiolucency and no response to EPT at the 1-year follow-up. In the CH group, 2 mature and 5 immature teeth failed at an average of a 17.0 ± 6.8-month follow-up because of pulp necrosis and infection with either clinical symptoms or radiographic signs.
Bakhtiar H et al. 2018 Iran (31)	Dental pulp response to RetroMTA after partial pulpotomy in permanent human teeth	Prospective clinical study	*n* = 22	18–32	Molar	Healthy pulp	Partial	EPT	Serial assessments: 1–8 weeks Loss to follow-up = 0	The electric pulp test revealed no sensitivity.
Bakhtiar H et al. 2017 Iran (32)	Human Pulp Responses to Partial Pulpotomy Treatment with TheraCal as Compared with Biodentine and ProRoot MTA: A Clinical Trial	Randomized clinical trial	*n* = 27	18–32	Molar	Healthy pulp	Partial	EPT	Serial assessments: 1–8 weeks Loss to follow-up = 0	Electric pulp test after 1 and 8 weeks revealed normal pulp response with no hypersensitivity.
Cem Güngör et al. 2007 Türkiye (33)	A retrospective evaluation of crown-fractured permanent teeth treated in a pediatric dentistry clinic.	Retrospective clinical study	*n* = 129	7–15	Incisor	Healthy pulp	Partial	EPT	Retrospective single assessment: 12 months	Vitality on admittance had a significant effect on the total time spent for definitive treatment. While percentage of vital teeth that were definitively treated in less than 6 months was 52.7%, the corresponding figure for non-vital teeth was 14.0.
Bjørndal et al. 2010 Denmark (34)	Treatment of deep caries lesions in adults: randomized clinical trials comparing stepwise vs. direct complete excavation, and direct pulp capping vs. partial pulpotomy.	Randomized clinical trial	*n* = 314	N/A	Incisor Premolar Molar	Reversible pulpitis	Partial	Cold test and EPT	Single assessment: 12 months Loss to follow-up = 29	Pulp vitality without apical radiolucency was 34.5% after partial pulpotomy; no significant difference vs direct pulp capping.
Simon et al. 2013 France (35)	Should pulp chamber pulpotomy be seen as a permanent treatment? Some preliminary thoughts.	Prospective clinical study	*n* = 17	7–54	Premolar Molar	Healthy pulp	Full	Cold test and EPT	Serial assessments: every 3 to 6 months up to 12 to 24 months Loss to follow-up = 0	The cold test was negative or the result was ambiguous in most cases and was not considered a reliable technique for pulp sensibility assessment. Most teeth responded to the EPT, but at different levels. A difference was noticed between the control and tested teeth, but this difference may have been a consequence of the type of coronal restoration or the weak electrical conductivity of the superimposed material.
Azimi et al. 2014 Iran (36)	Comparison of pulp response to mineral trioxide aggregate and a bioceramic paste in partial pulpotomy of sound human premolars: a randomized controlled trial.	Randomized controlled trial	*n* = 24	12–16	Premolar	Healthy pulp	Partial	Response to cold and hot stimulus	Serial assessments: 1 and 3 days, every once in 6 weeks	Amongst the 24 specimens, only five had sensitivity to cold stimuli, which did not exceed 1–2 s. All of these teeth were in the iRoot BP group. Four of these teeth were scored as mildly inflamed, whilst one had moderate inflammation. The chi-square test revealed a significant difference between the groups.
Bjørndal et al. 2017 Denmark (37)	Randomized clinical trials on deep carious lesions: 5-year follow-up.	Randomized clinical trial	*n* = 58	18–49	N/A	Healthy pulp	Partial	Cold test and EPT	Single assessment: 5 years Loss to follow up = 75	Only 3/27 partial pulpotomy cases retained pulp vitality without apical radiolucency.
Taha et al. 2017 Jordan (38)	Partial pulpotomy in mature permanent teeth with clinical signs indicative of irreversible pulpitis: A randomized clinical trial.	Randomized clinical trial	*n* = 50	20–52	Molar	Irreversible pulpitis	Partial	Cold test	Serial assessments: 6, 12, and 24 months Loss to follow up = 6	4 teeth were asymptomatic; however, the teeth gave a negative response to cold testing, and, radiographically, there was evidence of new periapical rarefaction.
Mehrvarzfar et al. 2018 Iran (39)	Clinical and histological responses of human dental pulp to MTA and combined MTA/treated dentin matrix in partial pulpotomy.	Prospective clinical study	*n* = 33	15–31	Molar	Healthy pulp	Partial	Cold test and EPT	Serial assessments: 1, 3, 7, 14, 21, 28, 35, and 42 days Loss to follow up = 4	The response to thermal tests at various intervals in each group was significantly different (*P* < 0.05). In other words, thermal sensitivity was more intense during the first 2 weeks in both groups and then reduced gradually.
Asgary et al. 2018 Iran (40)	Treatment outcomes of 4 vital pulp therapies in mature molars.	Randomized controlled trial	*n* = 32	12–75	Molar	Healthy pulp Reversible pulpitis Irreversible pulpitis	Full Partial	Cold test and EPT	Single assessment: 1 week Loss to follow up = 4	The response to the EPT at the 1-week follow-up was not significantly different among the groups (*P* > 0.05). However, the response to the cold test at the 1-week follow-up was significantly different among the groups (*P* < 0.05).
Eggmann et al. 2022 Switzerland (41)	Partial pulpotomy without age restriction: a retrospective assessment of permanent teeth with carious pulp exposure.	Retrospective clinical study	*n* = 111	18–85	Incisor Canine Premolar Molar	Healthy pulp Reversible pulpitis	Partial	Cold test	Retrospective assessment with a minimum follow-up of >1 year (Mean observation period: 3.1 ± 2.0 years) Loss to follow up = 27	The overall success rate of preserving tooth vitality was 89.1%.
Hussain et al. 2022 Bangladesh (42)	Outcome of Mineral Trioxide Aggregate Pulpotomy for mature permanent molars with symptoms indicative of irreversible pulpitis.	Quasi-experimental clinical study	*n* = 20	25–50	Molar	Irreversible pulpitis	Full	EPT	Serial assessments: 3, 6, and 12 months	At 3 and 6 months follow ups, all cases were positively responded to EPT.
Zhu et al. 2023 China (43)	Effect of full pulpotomy using a calcium silicate-based bioactive ceramic in adult permanent teeth with symptoms indicative of irreversible pulpitis: A retrospective study.	Retrospective clinical study	*n* = 81	18–72	Incisor Molar	Irreversible pulpitis	Full	Cold test and EPT	Serial assessments: 2 weeks-3, 6, and 12 months Loss to follow-up = 0	Six of the 81 teeth (7.4%) failed before the 12-month follow-up and RCT was performed. Among these 6 teeth, 3 exhibited severe cold stimuli pain and spontaneous pain at the 2-week follow-up, 2 had no response to the EPT with apical percussion pain and periapical rarefaction at the 3-month follow-up
Ramani et al. 2025 India (44)	Effect of lateral extent of pulp tissue removal on the outcome of partial pulpotomy for managing cariously exposed mature permanent molars with symptomatic irreversible pulpitis: A randomized clinical trial.	Randomized clinical trial	*n* = 81	18–45	Molar	Irreversible pulpitis	Restricted partial Extended partial	Cold test and EPT	Serial assessments: 6–12 months Loss to follow-up = 5	In restricted partial group, the positive responses to EPT and cold test were 92.3% and 89.7%. in extended partial group, the positive responses to EPT and cold test were 92.5% and 75%.
Zhu et al. 2024 China (45)	Full pulpotomy versus root canal therapy in mature teeth with irreversible pulpitis: a randomized controlled trial.	Randomized controlled trial.	*n* = 160	18–50	Molar	Irreversible pulpitis	Full	EPT	Serial assessments: 3, 6, and 12 months Loss to follow-up = 11	There were 5, 3, and 3 unresponsive teeth with EPT at 3-, 6- and 12- month follow-ups, respectively. Though the other teeth were all responsive, the values of EPT in postoperative were higher than preoperative.
Zheng et al. 2025 China (46)	Evaluation of iRoot BP Plus for pulpotomy in permanent anterior teeth with complicated crown fractures in pediatric patients: A prospective cohort study.	Prospective clinical study	*n* = 126	7–15	Incisor	Healthy pulp	Partial	Cold test	Serial assessments: 6–8 weeks, and 3, 6, and 12 months Loss to follow-up = not reported	>90% of treated teeth maintained pulp vitality and function without requiring root canal treatment.
Taha et al. 2024 Jordan (47)	Outcome and prognostic factors for partial and full pulpotomy in the management of spontaneous symptomatic pulpitis in carious mature permanent teeth: A randomized clinical trial	Randomized clinical trial	*n* = 200	11–61	Premolar Molar	Irreversible pulpitis	Partial Full	Cold test and EPT	Serial assessments: 6–12 months Loss to follow-up = 4	Ar 6 months, one tooth had a negative response to EPT and cold sensibility test without any clinical symptoms or radiographic changes. At 12 months, one tooth had a negative response to EPT and cold sensibility test without any clinical symptoms or radiographic changes.
Rana et al. 2025 India (48)	Evaluation of one- versus two-step technique on the outcome of mineral trioxide aggregate pulpotomy in mature permanent teeth with symptomatic irreversible pulpitis	Randomized clinical trial	*n* = 30	15–35	Molar	Irreversible pulpitis	Full	Cold test and EPT	Serial assessments: 3, 6, and 12 months Loss to follow-up = 0	Electric pulp tests were positive in all teeth, with 73.33% responding to cold testing.
Gilhotra et al. 2024 India (49)	Comparative evaluation of various biomaterials as pulpotomy agents in molars with symptomatic irreversible pulpitis: A randomized single-blinded single-center control trial	Randomized control trial	*n* = 80	14–60	Molar	Irreversible pulpitis	Full	Cold and heat tests	Serial assessments: 24 h, 1 and 4 weeks, and 3, 6, and 12 months Loss to follow-up = 7	All the experimental groups reported significant improvement in sensitivity to hot and cold and tenderness upon percussion at all intervals with Endosequence reporting maximum reduction in the number of patients with any clinical symptoms at 24 h of interval.
Jimenez-Martin et al. 2024 Spain (50)	Elective full pulpotomy in mature permanent teeth diagnosed with symptomatic irreversible pulpitis: a two years retrospective study	Retrospective clinical study	*n* = 43	11–76	Incisor Premolar Molar	Irreversible pulpitis	Full	Cold test	Serial assessments: variable times up to 24 months Loss to follow-up = 4	Only four teeth (10.3%) responded positively to the cold pulp sensitivity test: an upper second premolar, two lower first molars, and one upper first molar.
Zhang et al. 2025 China (51)	Clinical influencing factors of vital pulp therapy on pulpitis permanent teeth with 2 calcium silicate-based materials: A randomized clinical trial	Randomized clinical trial	*n* = 115	N/A	Incisor Premolar Molar	Reversible pulpitis Irreversible pulpitis	Partial	Cold and heat tests, EPT	Serial assessments: 3, 6, and 12 months Loss to follow-up = 11	At the 1-year recall session, the EPT test results were not significantly different between the 2 groups (*P* >.05). Tt is also noteworthy that almost 36.5% (19/52) of patients in the TH group had transient sensitivity to heat and cold at the 3-month visit and gradually relieved in subsequent observation, while in the BP group, the sensitivity was only found in the first 1 or 2 weeks.
Swetha et al. 2024 India (52)	The Outcome of GaAlAs Diode Laser (980 nm) pulpotomy in patients with symptomatic irreversible pulpitis assessed using CBCT—randomised controlled trial with an 18-month follow-up	Randomized controlled trial	*n* = 86	18–40	Molar	Irreversible pulpitis	Full	Cold test and EPT	Serial assessments: 6, 12, and 18 months Loss to follow-up = 33	Pulp sensibility testing was positive in 79% of teeth in conventional coronal pulpotomy group and 88% of teeth in laser coronal pulpotomy group.

### Characteristics of sources of evidence

The included evidence encompassed various study designs, primarily consisting of randomized controlled trials (RCTs), prospective clinical studies, retrospective clinical studies, and a quasi-experimental study. The studies were geographically diverse, conducted across multiple countries including India, USA, Germany, Singapore, Ireland, Jordan, Iran, China, Türkiye, Denmark, France, Switzerland, Bangladesh, and Spain. The sample sizes of the included studies varied considerably, ranging from 17 to 314 patients. The demographic age range of the patients was extremely broad, encompassing pediatric patients as young as 5 years old up to older adults aged 85. The anatomical distribution of the treated teeth included incisors, canines, premolars, and molars. Preoperative pulpal diagnoses investigated across the literature were categorized into healthy pulp, reversible pulpitis, and irreversible pulpitis.

### Synthesis of results

Regarding the diagnostic modalities used for post-operative pulp sensibility, 19 (54.3%) studies utilized a combination of EPT and cold tests. Eight (22.8%) studies relied exclusively on cold testing, while 7 (20%) employed EPT alone. Heat tests were the least utilized modality, appearing in only two (5.7%) studies (one combined with cold testing, and one combined with both cold testing and EPT). The follow-up timeframes for sensibility assessment demonstrated extreme heterogeneity across the literature. Evaluation periods ranged from short-term intervals of a few days or weeks (2–5 days, 1–8 weeks) to extended long-term follow-ups of 5 to 8 years. However, the vast majority of the studies (82.8%, 29/35) reported outcomes at 6-month interval.

In selected studies, the most commonly treated tooth type was molars (85.7%, 30/35), followed equally by premolars (31.4%, 11/35) and incisors (31.4%, 11/35). Regarding the anatomical distribution by dental arch, mandibular teeth were distinctly more frequently treated than maxillary teeth. Several clinical trials exclusively enrolled mandibular molars (16, 25). Among the studies that provided a detailed arch breakdown, mandibular teeth accounted for the vast majority of the treatments, with the exception of studies specifically focusing on complicated crown fractures where maxillary incisors predictably predominated (30). However, a subset of the trials only reported the general tooth type without explicitly stratifying the data into maxillary and mandibular arches. Only one study did not report any tooth type (2.9, 1/35). In addition, the studies were predominantly designed as randomized clinical trials (28.6%, 10/35,) and randomized controlled trials (25.7%, 9/35,), followed by prospective (8/35, 22.9%) and retrospective (7/35, 20.0%) clinical study designs. A single study employed a quasi-experimental design (2.9%, 1/35).

Among the studies included in this review conducted between 2007 and 2015 (11.4%, 4/35), no data were found regarding pulpotomy procedures performed with a diagnosis of irreversible pulpitis; the included studies during this period were limited to cases of healthy pulps or reversible pulpitis. Pulpotomy studies focusing on teeth diagnosed with irreversible pulpitis that met our specific eligibility criteria began to appear in the literature from 2017 to 2018 onwards. Between 2021 and 2025 (73.9%, 17/23), there was a marked increase in the included studies investigating this specific clinical indication, with full pulpotomy emerging as the predominant treatment approach for teeth diagnosed with irreversible pulpitis.

A discrepancy in positive response rates between EPT and thermal tests were found, the difference was heavily influenced by the extent of the pulpotomy procedure. Out of 19 clinical studies evaluating full pulpotomies, 13 (68.4%) utilized both cold test and EPT (16, 27, 35, 40, 48, 52), while 3 (15.8%) used only cold test (46, 49, 50) and 3 (15.8%) used only EPT (29, 42, 45). Among the studies that performed both cold test and EPT to compare the difference, 6 (46.1%) highlighted that a significantly higher rate of positive responses was obtained with EPT, while 7 (53.9%) of them presented non-comparable results. On the other hand, among the 18 studies assessing partial pulpotomy, 8 (44.4%) employed a combination of cold test and EPT, whereas 6 (33.3%) relied solely on cold testing, and 4 (22.2%) exclusively on EPT. Notably, only 2 (25%) of these studies comparatively evaluated the responses between the cold test and EPT, both reporting a higher rate of positive responses with EPT. In rare instances, complete loss of sensibility was observed; one randomized clinical trial reported no response to either EPT or cold testing throughout a 1-year period following full pulpotomies (27).

Specifically, studies involving pediatric patients aged 5–15 years and those investigating crown fractures in incisors predominantly relied on a single sensibility test, using either EPT or the cold test alone (11.4, 4/35). In contrast, studies targeting adult populations and predominantly involving mature permanent teeth (88.6, 31/35) demonstrated a clear shift toward the combined use of the cold test and EPT (61.3%, 19/31) for postoperative pulp sensibility assessment.

## Discussion

The available evidence on postoperative pulp sensibility assessment following partial and full pulpotomy spans several decades, during which substantial changes have occurred in clinical methods, materials, diagnostic terminology, and outcome reporting. Earlier studies frequently omitted postoperative pulp sensibility testing or relied on non-standardized methods, whereas more recent clinical studies have increasingly adopted standardized guidelines (53, 54). The primary objective of this scoping review was to systematically map the clinical methods, diagnostic modalities, and follow-up timeframes used to assess pulp sensibility following permanent tooth pulpotomy. The synthesized evidence revealed a profound lack of methodological standardization across the literature. While neural sensibility tests, primarily the EPT and cold tests remain the diagnostic cornerstones, the chronologic intervals for assessment present extreme heterogeneity, ranging from 24 h to 8 years post-operatively. A significant finding of this review is the profound under-reporting of crucial operational details in the primary literature. Important methodological parameters, such as the specific cold-testing agent, stimulus duration, isolation techniques, site of application, use of a control tooth, EPT conducting medium, definition of a positive response threshold, and examiner calibration, were frequently omitted. The absence of these critical data limits the review strictly to the observation of broad test modalities and assessment timeframes, highlighting an urgent need for comprehensive reporting standards.

The earliest studies (2007–2015) were largely confined to healthy pulp and reversible pulpitis cases, generally relying on a single test (most often EPT or cold). From 2017 onwards, accompanied by a paradigm shift in pulp biology and the advent of bioactive biomaterials, there was a clear transition towards multimodal assessment methods (8). This reflects updated position statements from major endodontic organizations (9, 10, 55), which explicitly advocate for comprehensive follow-up evaluations combining clinical, radiographic, and sensibility testing to accurately ascertain the survival of the retained pulp tissue.

A major finding of this review is the observed discordance between EPT and cold tests, particularly following full pulpotomies. Multiple studies consistently reported a higher proportion of positive responses to EPT compared to cold stimuli, which frequently yielded negative or ambiguous results despite clinical and radiographic success (16, 28). In this sense, the physiological basis of pulp sensibility testing is important when interpreting postoperative responses following pulpotomy. Cold testing primarily relies on the hydrodynamic mechanism, whereby rapid thermal contraction of dentinal fluid generates mechanical forces within dentinal tubules and activates mechanosensitive A-delta fibers at the pulp–dentin interface. These myelinated fibers have a relatively low activation threshold and are responsible for the sharp, short-lasting pain typically elicited by cold stimuli. In contrast, heat stimulation is more likely to activate slowly conducting, unmyelinated C-fibers, which are associated with dull, poorly localized, and often lingering pain and may become particularly responsive in the presence of pulpal inflammation (56). On the other hand, EPT differs fundamentally from thermal testing because it delivers an electrical stimulus that directly depolarizes excitable pulpal nerve fibers, predominantly A-delta fibers, without depending on dentinal fluid movement. Therefore, EPT and thermal tests assess neural responsiveness through different physiological pathways rather than measuring the biological vitality of the pulp itself (16). Following full pulpotomy, removal of the coronal pulp and odontoblastic layer may alter the dentin–pulp sensory interface and reduce the efficiency of thermal stimulus transmission. In addition, the thickness and thermal insulating properties of the restorative material covering the radicular pulp may further attenuate the temperature change reaching the remaining neural tissue. In contrast, an electrical stimulus may be transmitted more effectively through the restored tooth and reach radicular nerve fibers, potentially explaining the higher proportion of positive EPT responses observed after full pulpotomy (29). Thus, the discordance between EPT and thermal testing may reflect differences in stimulus transmission and neural activation rather than a true difference in the biological status of the remaining pulp tissue.

Another confounding factor when interpreting the results of the included studies is the lack of standardization among electric pulp testers. Commercially available EPT devices operate with varying electrical parameters, including differences in current intensity, voltage, pulse duration, frequency or interval settings, and the rate of stimulus increase. These instrumental variations might directly influence sensory perception thresholds, thereby producing differing response patterns among patients and across clinical trials. A notable limitation within the current literature is the heterogeneity of clinical parameters, which complicates the interpretation of pulp sensibility tests. Various confounding variables, such as the etiology of pulpal injury (caries vs. trauma), root maturity, baseline diagnosis, the extent of the pulpotomy, hemostasis protocols, and the specific biomaterials used, can significantly influence EPT and cold-test responses. Furthermore, differences in restorative configurations and material thicknesses play a critical role in thermal conductivity.

The transient nature of post-operative sensibility further complicates the assessment of pulpal health. Studies indicated that heightened sensitivity to thermal stimuli is often most intense during the initial 1 to 2 weeks following partial pulpotomy (39, 46). This early thermal hypersensitivity is likely a manifestation of the initial acute inflammatory response and subsequent pulpal healing, rather than an indicator of irreversible pathosis. If sensibility testing is conducted too early, these hyper-reactive responses can be easily misinterpreted by clinicians as persistent irreversible pulpitis, potentially leading to unnecessary pulpectomy. Therefore, definitive conclusions regarding pulp survival should not be based on immediate post-operative testing. Most importantly, neither a positive sensibility response nor the presence of vascularity alone equates to overall pulpal health or treatment success. True clinical and radiographic success requires a complete absence of clinical symptoms and periapical pathosis over time, representing definitive tissue healing. Therefore, the neural tests evaluated in this review serve merely as proxies for pulpal survival and neurogenesis, rather than definitive indicators of overall treatment success.

While authors acknowledge that case reports and series often provide valuable clinical insights, they were excluded, alongside review articles, editorials, conference abstracts, and non-peer-reviewed publications, to maintain a consistent focus on structured clinical studies with higher levels of evidence. Furthermore, it is important to recognize that the interpretation of EPT and cold-test findings is inherently influenced by numerous confounding variables, including baseline diagnosis, root maturity, hemostasis protocols, and restorative material thickness. The inconsistent reporting of these critical variables across primary studies limits the ability to draw definitive causal conclusions and highlights the need for more rigorous reporting standards in future clinical studies. Future research should prioritize the development of standardized postoperative pulp sensibility assessment methods following pulpotomy, including consensus on the most appropriate sensibility tests, assessment intervals, and interpretation criteria to improve the consistency and comparability of clinical outcomes. In addition, multicenter randomized clinical trials with extended follow-up periods are needed to better understand the long-term recovery of pulpal sensibility and to determine how factors such as patient age, stage of root development, preoperative pulp diagnosis, pulpotomy technique, and restorative material influence postoperative responses. Furthermore, future studies should investigate the clinical applicability and diagnostic performance of objective vascular-based vitality assessment methods in combination with sensibility tests.

## Conclusion

EPT and cold tests remain the standard diagnostic tools used in clinical studies, with EPT frequently demonstrating a higher proportion of positive responses, depending on the specific clinical applications and the extent of the pulpotomy procedure. Full-pulpotomy studies report that EPT elicits positive responses more frequently than cold testing. However, the diagnostic and prognostic implications of this discordance currently remain uncertain. To improve the consistency of outcome reporting and facilitate comparisons among future vital pulp therapy studies, there is a clear need for the development of standardized clinical guidelines.
