# Supplementary material for: Pulp sensibility assessment following permanent tooth pulpotomy: a scoping review of clinical studies
Source: Front Dent Med. 2026 Sep 22;7:1957091. doi: 10.3389/fdmed.2026.1957091 (PMC13639861; doi:10.3389/fdmed.2026.1957091)
Supplement: Supplementary file 1 [file Supplementaryfile1.docx]

**Suppl. Table 1.** Mapping of specific sensibility test modalities in the 35 included clinical studies

| **Author, year, country** | **Cold Test (Agent)** | **Heat Test**  **(Agent)** | **EPT**  **(Device)** | **Isolation method** | **Site of stimulus application** | **Duration of stimulus application** | **Control tooth** | **Examiner calibration** | **Response threshold** |
| --- | --- | --- | --- | --- | --- | --- | --- | --- | --- |
| Rechithra et al. 2023  India (62) | Endofrost | - | Kerr Vitality Scanner | NR | NR | NR | NR | NR | NR |
| Shallal-Ayzin et al.  2021  USA (22) | EndoIce | - | Kerr Vitality Scanner | NR | NR | NR | NR | + | NR |
| Petrovic et al.  2023  Germany (23) | +  (Agent NR) | - | - | NR | NR | NR | NR | NR | NR |
| Chua et al.  2023  Singapore (24) | EndoIce | - | Kerr Vitality Scanner | NR | NR | NR | NR | NR | NR |
| Careddu et al.  2021  Ireland (13) | Endofrost | - | - | NR | NR | NR | NR | NR | NR |
| Rechithra et al. 2021  India (25) | +  (Agent NR) | - | +  (Agent NR) | NR | NR | NR | NR | NR | NR |
| Al-Batayneh et al.,  2022  Jordan (26) | EndoIce | - | - | NR | NR | NR | NR | NR | NR |
| Mishra et al.  2024  India (27) | EndoIce | - | Kerr Vitality Scanner | NR | NR | NR | NR | NR | NR |
| Aravind et al.  2022  India (16) | Endofrost | - | Kerr Vitality Scanner | EPT: Cotton rolls | Midway between the gingival margin and the occlusal edge of the buccal surface | 5 s (cold) | Contralateral tooth (tested with treated tooth) | NR | NR |
| Jassal et al.  2023  India (28) | Endofrost | - | Digitest II | NR | NR | NR | NR | NR | NR |
| Asgary et al.  2022  Iran (6) | +  (Agent NR) | - | +  (Agent NR) | NR | NR | NR | NR | NR | NR |
| Kazemipoor et al.  2018  Iran (29) | - | - | Gentle-pulse | NR | NR | NR | NR | NR | NR |
| Rao et al.  2020  China (30) | - | - | +  (Agent NR) | NR | NR | NR | NR | NR | NR |
| Bakhtiar H et al.  2018  Iran (31) | - | - | +  (Agent NR) | NR | NR | NR | NR | NR | NR |
| Bakhtiar H et al.  2017  Iran (32) | - | - | +  (Agent NR) | NR | NR | NR | NR | NR | NR |
| Güngör et al.  2007  Türkiye (33) | - | - | +  (Agent NR) | NR | NR | NR | NR | NR | NR |
| Bjørndal et al.  2010  Denmark (34) | EndoIce | - | Kerr Vitality Scanner | NR | NR | NR | NR | NR | NR |
| Simon et al.  2013  France (35) | Endocold | - | Digitest | EPT: Cotton roll | EPT: Buccal surface | NR | Contralateral tooth or homologous tooth | NR | NR |
| Azimi et al.  2014  Iran (36) | +  (Agent NR) | +  (Agent NR) | - | NR | NR | NR | NR | NR | NR |
| Bjørndal et al.  2017  Denmark (37) | +  (Agent NR) | - | +  (Agent NR) | NR | NR | NR | NR | NR | NR |
| Taha et al.  2017  Jordan (38) | EndoIce | - | - | NR | NR | NR | NR | NR | NR |
| Mehrvarzfar et al.  2018  Iran (39) | +  (Agent NR) | - | +  (Agent NR) | NR | NR | NR | NR | NR | NR |
| Asgary et al.  2018  Iran (40) | +  (Agent NR) | - | +  (Agent NR) | NR | NR | NR | NR | NR | NR |
| Eggmann et al.  2022  Switzerland (41) | +  (Agent NR) | - | - | NR | NR | NR | NR | NR | NR |
| Hussain et al.  2022  Bangladesh (42) | - | - | +  (Agent NR) | NR | NR | NR | NR | NR | NR |
| Zhu et al.  2023  China (43) | +  (Agent NR) | - | +  (Agent NR) | NR | NR | NR | NR | NR | NR |
| Ramani et al.  2025  India (44) | Endofrost | - | C-Pulse | NR | NR | NR | NR | NR | NR |
| Zhu et al.  2024  China (45) | - | - | Denjoy | NR | NR | NR | intact teeth of contralateral homonymous, opposite jaw homonymous, or adjacent | NR | NR |
| Zheng et al.  2025  China (46) | +  (Agent NR) | - | - | NR | NR | NR | NR | NR | NR |
| Taha et al.  2024  Jordan (47) | EndoIce | - | +  (Agent NR) | NR | NR | NR | NR | NR | NR |
| Rana et al.  2025  India (48) | +  (Agent NR) | - | +  (Agent NR) | NR | NR | NR | NR | NR | NR |
| Gilhotra et al.  2024  India (49) | Endofrost | Gutta-percha sticks | - | NR | NR | NR | NR | NR | NR |
| Jimenez-Martin et al.  2024  Spain (50) | +  (Agent NR) | - | - | NR | NR | NR | NR | NR | NR |
| Zhang  et al.  2025  China (51) | +  (Agent NR) | +  (Agent NR) | +  (Agent NR) | NR | NR | NR | Compared with control tooth | NR | EPT: <5 / 5–10 / >10 |
| Swetha et al. 2024  India (52) | EndoIce | - | +  (Agent NR) | NR | NR | NR | NR | NR | NR |

EPT: Electric Pulp Test; (+): Indicates the modality was explicitly reported and used in the study for post-operative follow-up. NR: not reported in the full text.
